# New insights into the role of adipocytes in pancreatic cancer progression: paving the way towards novel therapeutic targets

**DOI:** 10.7150/thno.82911

**Published:** 2023-07-03

**Authors:** Yu-Chun Lin, Ya-Chin Hou, Hao-Chen Wang, Yan-Shen Shan

**Affiliations:** 1 Institute of Clinical Medicine, College of Medicine, National Cheng Kung University, Tainan 704, Taiwan.; 2 Department of Clinical Medicine Research Center, National Cheng Kung University Hospital, College of Medicine, National Cheng Kung University, Tainan 704, Taiwan.; 3 Division of General Surgery, Department of Surgery, National Cheng Kung University Hospital, College of Medicine, National Cheng Kung University, Tainan 704, Taiwan.; 4 Medical Imaging Center, Innovation Headquarter, National Cheng Kung University; Tainan 704, Taiwan.

**Keywords:** pancreatic cancer, tumor microenvironment, adipocyte dysfunction, fat loss, adipokines, trans-differentiation

## Abstract

Pancreatic cancer (PC) remains one of the most lethal malignancies across the world, which is due to delayed diagnosis and resistance to current therapies. The interactions between pancreatic tumor cells and their tumor microenvironment (TME) allow cancer cells to escape from anti-cancer therapies, leading to difficulties in treating PC. With endocrine function and lipid storage capacity, adipose tissue can maintain energy homeostasis. Direct or indirect interaction between adipocytes and PC cells leads to adipocyte dysfunction characterized by morphological change, fat loss, abnormal adipokine secretion, and fibroblast-like transformation. Various adipokines released from dysfunctional adipocytes have been reported to promote proliferation, invasion, metastasis, stemness, and chemoresistance of PC cells via different mechanisms. Additional lipid outflow from adipocytes can be taken into the TME and thus alter the metabolism in PC cells and surrounding stromal cells. Besides, the trans-differentiation potential enables adipocytes to turn into various cell types, which may give rise to an inflammatory response as well as extracellular matrix reorganization to modulate tumor burden. Understanding the molecular basis behind the protumor functions of adipocytes in PC may offer new therapeutic targets.

## 1. Introduction

Pancreatic cancer (PC) is one of the most lethal malignancies in the world 1. Despite advancements in treatments, the 5-year survival rate remains extremely poor. PC is characterized by a highly heterogeneous tumor microenvironment (TME). Apart from PC cells, the TME contains various nonmalignant cell types including vascular endothelial cells, fibroblasts, adipocytes, stellate cells, mesenchymal stem cells (MSCs), and various immune cells. The complex interactions within the pancreatic TME give rise to multiple pathological phenomena, such as chronic inflammation, tissue fibrosis, and even immune escape, which allow PC cells to survive various therapies and disseminate to distant organs. 2-4. During tumorigenesis, immune cells, particularly macrophages, dendritic cells (DCs), monocytes, and T cells, are recruited to the tumor site to fight against malignant cells through their cytotoxic functions and secretion of pro-inflammatory cytokines. However, chronic inflammation in the TME can impair immune cells or even induce their transformation into a pro-tumor phenotype 5, 6. Additionally, cancer-derived factors, such as transforming growth factor beta (TGF-β), can activate pancreatic stellate cells (PSCs) and fibroblasts, facilitating the deposition of extracellular matrix (ECM) components and the excessive secretion of cytokines and chemokines 7. These processes contribute to the development of a desmoplastic TME 8-10. The hyperplastic stroma in the pancreatic tumor restricts vascularization, creating a physical barrier to anti-tumor reagents and a hypoxic environment that promote cancer development. Besides facilitating tumor progression, the severe cytokine storm generated by the TME can also lead to systemic defects, including adipose dysfunction 11-13.

Adipose tissue regulates energy homeostasis through its lipid storage and endocrine functions 14, 15. However, pro-inflammatory cytokines derived from the TME can perturb lipid metabolism and adipokine secretion, promoting cancer development in various ways 15. Previous research has established a strong association between adipose tissue and PC progression, including a higher incidence of tumor metastasis and chemoresistance in obese PC patients 16-19. Adipose tissue loss and altered circulating lipid profiles are also observed in both PC animal models and clinical studies 20-22. Additionally, even among PC patients with normal body mass index (BMI) and average blood sugar levels, the presence of peripancreatic fat infiltration portends a high risk of locoregional recurrence and poor overall survival 23-25. Intrapancreatic fat deposition is also associated with increased levels of pro-inflammatory cytokines in the circulation, which interferes with the immune response and reinforces the development of *Kras*-driven pancreatic tumorigenesis 26, 27. These findings identify the significant role of adipose tissue in the development of PC, underscoring the urgent need to better understand the underlying mechanisms for developing therapeutic strategies. In this article, we review recent studies about the molecular mechanisms by which adipose tissue promotes PC development, including nutrient support, endocrine function, and the effects of its plasticity on PC cells with surrounding TME.

## 2. Characteristics, functions, and development of normal adipose tissue

### 2.1. Composition of adipose tissue

Adipose tissue is a specialized connective tissue containing two major fractions, lipid-rich adipocytes, and the stromal vascular fraction. Adipocytes store body fat in the form of triglycerides within lipid droplets in their cytoplasm. The stromal vascular fraction consists of various cell types, including endothelial cells, preadipocytes, adipose-derived mesenchymal stem cells (ADSCs), and immune cells. The remarkable heterogeneity of adipose tissue contributes to its multifunctionality and cooperative role in modulating energy balance. Both fractions of adipose tissue are surrounded by the ECM, which serves as a scaffold supporting multiple biological processes, such as cell adhesion, signal transduction, and the formation of new blood vessels 28. Endothelial cells within the adipose tissue also play a vital role in promoting angiogenesis through their proliferation and migration, ensuring an adequate nutrient supply and guiding immune cells for tissue repair 29, 30. The tight coordination between the ECM and endothelial cells is closely associated with the expansion of adipocyte precursors and the overall adipose mass, allowing adipose tissue greater expandability and adaptability in response to diverse external stimuli and systemic nutritional status. Apart from non-adipocyte cell types, different categories of adipocytes also exhibit distinct functions in regulating energy homeostasis.

### 2.2. Function and category of adipocytes

Adipose tissue modulates energy homeostasis by lipid reservation and endocrine secretion 15. According to functional properties, adipose tissue can be divided into three types, namely white adipose tissue (WAT), beige adipose tissue, and brown adipose tissue (BAT). WAT accounts for the largest proportion of adipose tissue and is characterized by single large lipid droplets in the cytoplasm.

**Figure 1:**
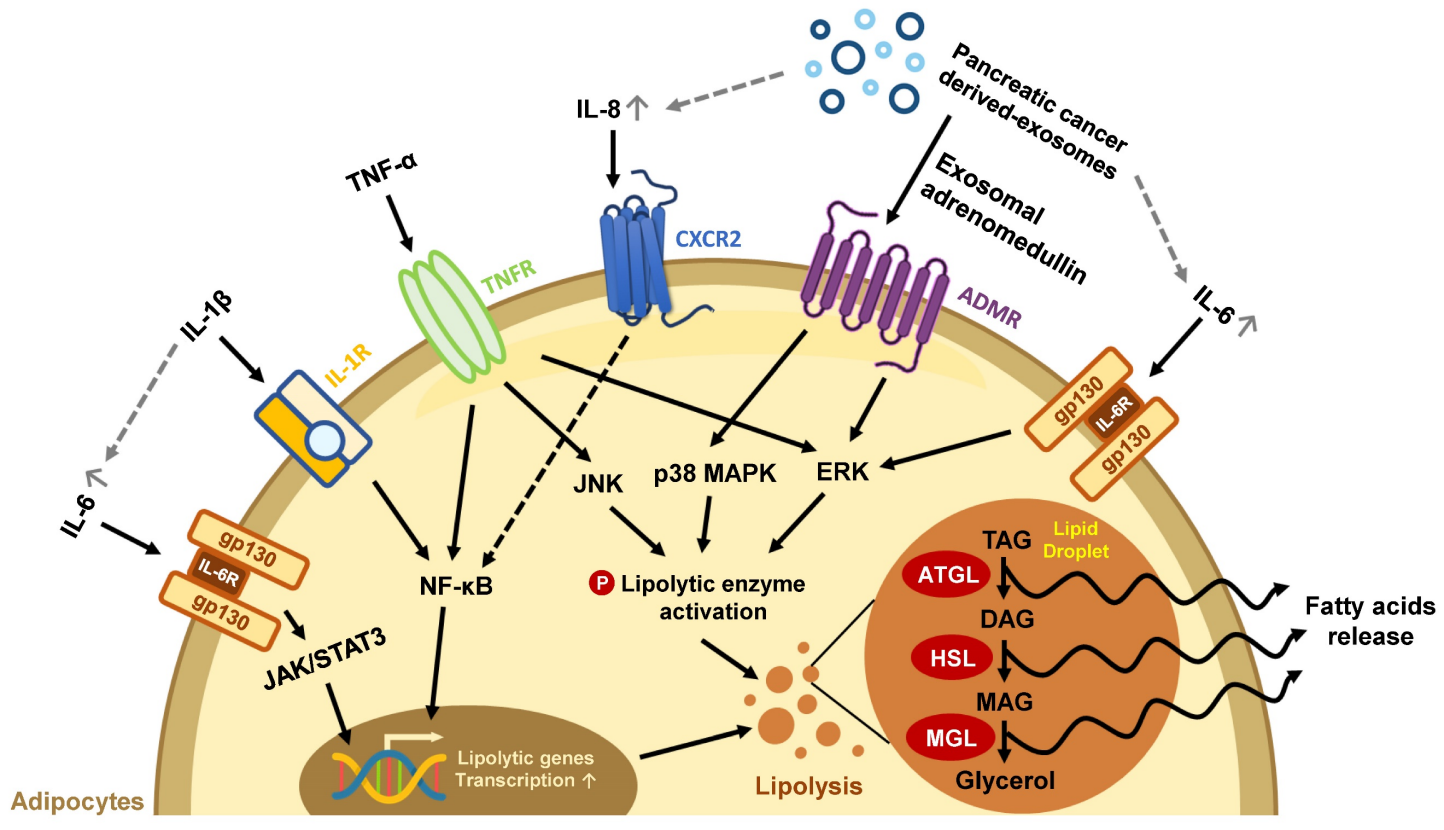
** The effects of secretion factors in pancreatic TME on lipid loss in mature adipocytes.** Exosomes and pro-inflammatory cytokines released from pancreatic TME trigger excessive lipolysis in mature adipocytes, leading to a decline in lipid storage. The gray dotted line represents the exosome/cytokine involved in IL-6 or IL-8 production.

WAT governs metabolic actions in distant organs by releasing numerous adipokines into the circulation 31, while fat storage in WAT is subject to lipid mobilization in response to environmental signals. Nutrients taken up from diet are transported to the liver and converted into lipids through *de novo* lipogenesis. These newly synthesized fats are then delivered and incorporated into WAT for storage by lipoproteins. To enhance energy storage capacity, adipocytes change through two mechanisms. Firstly, they can incorporate additional lipids content into their lipid droplets leading to an enlargement of their volume, a process known as adipocyte hypertrophy. Secondly, adipocytes can also increase in number by recruiting precursor cells and promoting their proliferation, referred to as hyperplasia. These two processes collectively contribute to the overall growth of adipose tissue mass. In contrast, systemic energy demands, energy deficiency, or catabolic stimulation serve as external stimuli that initiate the process of lipolysis, whereby stored lipid droplets within adipocytes undergo breakdown (**Figure 1**). Perilipin plays as a gatekeeper in the regulation of lipolysis by forming a complex with CGI-58 on the surface of lipid droplets. When lipolysis is activated, Perilipin undergoes phosphorylation, leading to the dissociation of CGI-58 from the complex. This action facilitates the subsequent binding of CGI-58 to adipose tissue triglyceride lipase (ATGL), enabling the breakdown of triglycerides (TGs) into diglycerides (DGs) and free fatty acids (FFAs). Phosphorylated Perilipin also recruits hormone-sensitive lipase (HSL) to the surface of lipid droplets, working together to break down DGs into FFAs and monoglycerides (MGs). Monoglyceride lipase (MGL) manages the final step in the lipolysis process that breaks down MGs into glycerol and FFAs 32. Released FFAs can further be plunged into β-oxidation to generate adenosine triphosphate (ATP) for energy production.

Unlike WAT, BAT is characterized by the presence of multiple small lipid droplets and high levels of mitochondria. BAT has a substantial contribution to thermoregulation, allowing the body to adjust and maintain its temperature in response to various physical conditions. The high expression of mitochondria uncoupling protein 1 (UCP1) enables BAT to specialize in heat generation. UCP1 acts as a proton pump that balances the ion gradient across the mitochondrial inner membrane during oxidative phosphorylation. Instead of ATP synthesis, this process converts transmembrane potential into heat production 33. Beige adipocytes residing in WAT originate from the same mesenchymal lineage as WAT, yet harbor a similar phenotype to BAT, possess multilocular lipid droplets, and abundant mitochondria capable of thermogenesis 34. Several external stimuli such as cold exposure 35, adrenergic stimulation 36, and nutritional manipulation or pharmacological treatments 37, 38, have been demonstrated to induce biogenesis and thermogenesis in beige adipocytes, leading to increased heat production and energy expenditure. Beige adipocytes can also protect against obesity and type 2 diabetes by increasing lipolysis, fatty acid oxidation, glucose uptake, and insulin sensitivity with reduced adiposity 39, 40. While each of these three types of adipocytes has its unique functions, they are all essential in maintaining energy balance.

### 2.3. Development of different types of adipocytes

Multipotent MSCs have been suggested as a major source of adipocyte generation, in which numerous critical pathways are involved, such as the TGF-β/bone morphogenic protein (BMP) pathway, Wingless-related integration site (Wnt) signaling, fibroblast growth factors (FGFs) signaling, Hedgehogs signaling, and Notch signaling 41. The differentiation direction of adipocytes depends on the expression of myogenic Factor-5 (*Myf5*). Brown adipocytes are derived from the *Myf5^+^* lineage, sharing the same transcriptional profiles as skeletal muscle cells. In contrast, beige and white adipocytes are developed from *Myf5^-^* progenitor cells. A transcriptional complex composed of BMP-7 signals, PR domain containing 16 as well as CCAAT/enhancer-binding protein (C/EBP) beta drives* Myf5*^+^ precursors into a thermogenic program with co-activation of peroxisome proliferator-activated receptor gamma (PPAR-γ) and PPAR-γ co-activator 1 alpha (PGC-1α), facilitating the formation of brown adipocytes. Partial *Myf5^-^* progenitors also undergo a thermogenic process, but they transform into beige-adipocytes with specific genes expression like CD137, transmembrane protein 26, and T-box 1 34. The other *Myf5^-^* population turns into white pre-adipocytes through BMP2 and BMP4 signaling 42. PPAR-γ as well as C/EBP family proteins further promote adipogenesis by upregulating lipogenic genes. In the late stage of white adipocyte differentiation, lipid accumulation, and high adipokine secretion, especially leptin and adiponectin can be observed 43. In addition to d*e novo* differentiation, beige adipocytes can be also transdifferentiated from white adipocytes under certain conditions like cold exposure, β3-adrenergic signals triggered by the sympathetic neuron system, and chronic inflammation in various pathological processes 44. The white-beige adipocyte transition known as “fat browning” turns the fat storage into the fuel for thermogenesis, leading to energy expenditure and loss of lipid stores. This shows the multidirectional development of adipocytes in response to different environmental stimuli.

## 3. Adipose tissue alteration in PC development

Exogenous signals and stimuli can greatly affect adipocyte functions and features. During cancer progression, uncontrolled cancer cell proliferation brings high nutrient demands that break normal energy balance 45, 46; numerous pro-inflammatory cytokines released from the TME trigger catabolic reactions through different pathways in adipose tissue (**Figure 1**). Inflamed adipose tissue may also undergo morphological transition to other cell types which results in the loss of their original function. In this section, we sum up the findings of how PC induces functional defects in adipose tissue, and how the latter conforms to promote PC growth.

### 3.1. Impaired energy reservation

#### 3.1.1. Loss of lipid storage during pancreatic tumor progression

Abnormal lipid metabolism in adipocytes has been found in the development of various cancers, including PC 47-49. Pancreatic tumors are characterized by their massive stroma which gives rise to a hypoxic environment. In this state, PC cells cannot synthesize fatty acids by *de novo* lipogenesis; instead, they take up exogenous FFAs to maintain lipid homeostasis under hypoxic stress 50. For example, exosomes secreted from PC cells carry adrenomedullin and can bind to the adrenomedullin receptors on adipocytes, activating lipolysis enzyme HSL via p38 mitogen-activated protein kinases (MAPKs) and extracellular signal‑regulated protein kinase (ERK) 1/2 dependent phosphorylation to turn on lipolysis and release FFAs 51. PC cells can also cause lipid breakdown directly through cytokine secretion. Numerous pro-inflammatory cytokines such as interleukin (IL)-6, IL-1β, IL-8, and TNF-α in the pancreatic TME have been associated with advanced disease status in clinical studies and can trigger a catabolic response in adipocytes 13. IL-6 activates its downstream Janus kinase (JAK)/signal transducer and activator of the transcription (STAT) pathway and the ERK pathway to increase lipolytic gene expression and enzyme activity 52. IL-1β derived from pancreatic tumors stimulates IL-6 production from adipocytes, which aggravates autocrine lipid breakdown in adipocytes 53. TNF-α activates lipolytic enzymes through stimulating ERK and Jun N-terminal kinase (JNK) kinase activity 54. Additionally, both IL-1β and TNF-α promote NF-κB nuclear translocation and increase lipolytic gene transcription (**Figure 1**) 55-57. PC cells-derived exosomes also enhance the release of inflammatory cytokines IL-6, IL-8, and C-C motif chemokine ligand 2 (CCL2) and promote lipolysis in adipocytes. IL-6 blockade abrogates PC-exosome-induced lipolysis 58, supporting the important role of inflammatory cytokines in lipolysis.

In addition to upregulated lipolysis, WAT may undergo beige adipocyte-transition during chronic inflammation, leading to inefficient energy use through UCP1 expression. IL-6 and parathyroid-hormone-related protein, a hormone frequently upregulated in PC with *Kras* mutation 59, have been reported to induce fat browning during PC progression 52, 60, 61. Both lipolysis and fat browning in mature adipocytes increase lipid consumption, leading to the loss of adipocytes' energy storage function.

#### 3.1.2. Energy resources from adipocytes enhance pancreatic tumor development

PC patients with obesity suffer increased tumor burden and faster disease progression compared with those without obesity 62, indicating that enriched lipid depots may exacerbate tumor growth. Another study also mentioned that in KC (*fElas^CreERT^; Kras^G12D/+^*) transgenic mice, a high-fat diet increased the activity of Kras, a substantial oncogenic factor in PC development, while the numbers of both PanIN and PC tumors were increased during the intervention 63, 64. Furthermore, a high-fat diet enhanced distant metastasis and fat invasion of PC in KPC (*Pdx1-Cre; LSL-Kras^G12D^; Trp53^R172H/+^*) transgenic mice 65, which was also confirmed by direct fatty acid treatment *in vitro*. The above studies support the idea that lipid resources derived from adipose tissue promote PC progression. Lipids not only serve as substrates for ATP production, but also play central roles in cell membrane construction, signaling transduction, and posttranslational protein modification, all of which are necessary for tumor expansion. 66, 67. Fatty acids released from adipocytes promote PC development in multiple ways. PC cells treated with primary adipocyte-derived conditioned medium containing oleic, linoleic, and palmitoleic acids, acquired extended lipid storage in their cytoplasm, and demonstrated increased migration and invasion abilities. These pro-tumor effects are positively correlated with the concentration of fatty acids 65. PC cells take up exogenous FFAs through surface fatty acid translocase-CD36, shutting down this channel with CD36 inhibitors suppresses the enhancement of cancer cell migration by adipocyte condition medium 65. These FFAs are usually stored as lipid droplets in PC cells to prevent lipotoxicity 49, 68 and reactive oxygen species (ROS) toxicity in hypoxic TME 69.

Besides tumor cells, noncancerous cells in the TME are also affected by abnormal lipid metabolism during cancer progression. PC has a highly immunosuppressive TME where immune cells fail to destroy tumor cells or even transit into a pro-tumor phenotype, exemplified by tumor-associated macrophages (TAMs). Under long-term stimulation with cytokines such as IL-4, IL-10, and IL-13 in the TME, cytotoxic M1 macrophages are polarized into the M2 phenotype with anti-inflammation, pro-angiogenic, and tissue remodeling abilities to promote tumor growth 70, 71. This polarization process heavily relies on fatty acid oxidation (FAO) for energy supply, which can be supported by extra fatty acids from adipose tissue during tumor progression 72, 73.

CD8^+^ T lymphocytes recognize specific antigenic peptides presented on the tumor cells by human leukocyte antigen class I and are activated to kill malignant cells through secreting cytotoxic granules or cytokines like interferon and TNF 74. For prompt energy support, glycolysis is engaged in activated CD8^+^ T cells 75. However, glycolytic genes are downregulated in infiltrating CD8^+^ T cells in a lipid-rich environment 76, 77. The uptake and accumulation of long-chain fatty acids in the cytoplasm can lead to lipotoxicity and thus cell death of CD8^+^ T cells 77. The increased concentration of fatty acids in the TME not only benefits PC cell growth but also disturbs anti-cancer immunity.

Adipocytes can also secrete glutamine to preserve PC cell proliferation in nutrient-poor conditions 78, 79. PC cells convert glutamine into α-ketoglutarate that can fuel the tricarboxylic acid cycle to turn on multiple biogenesis processes, further indicating the significant role of adipocytes in PC development 78. Apart from abnormal catabolic reactions of adipose tissue during cancer progression, the extraordinary release of adipose-derived factors also contributes to pancreatic tumor growth.

### 3.2. Defective endocrine functions

Adipose tissue produces a variety of hormones called adipokines to regulate the metabolic processes throughout the body. However, adipocyte functions can be impaired under pathological conditions. In obesity, adipocytes undergo hypertrophy and hyperplasia, leading to excessive secretion of cytokines and the recruitment of immune cells, which results in low-grade inflammation, and further compromising adipocyte function. This is accompanied by elevated serum levels of leptin, resistin, hepatocyte growth factor (HGF), and TNF-α, along with the decreased release of adiponectin, insulin-like growth factors (IGF), and type two cytokines, which hinder systemic catabolic pathways, insulin sensitivity, and immune regulation 80. In the context of obesity and cancer, alterations in immune cell populations have been observed. The numbers of M1 macrophages are reduced 81, while inhibitory dendritic cells 82, suppressive T cells 83, and dysfunctional T cells 76 are often found in higher abundance. These observations suggest that secretory factors derived from adipose tissue may play a role in modulating the immune system.

**Figure 2:**
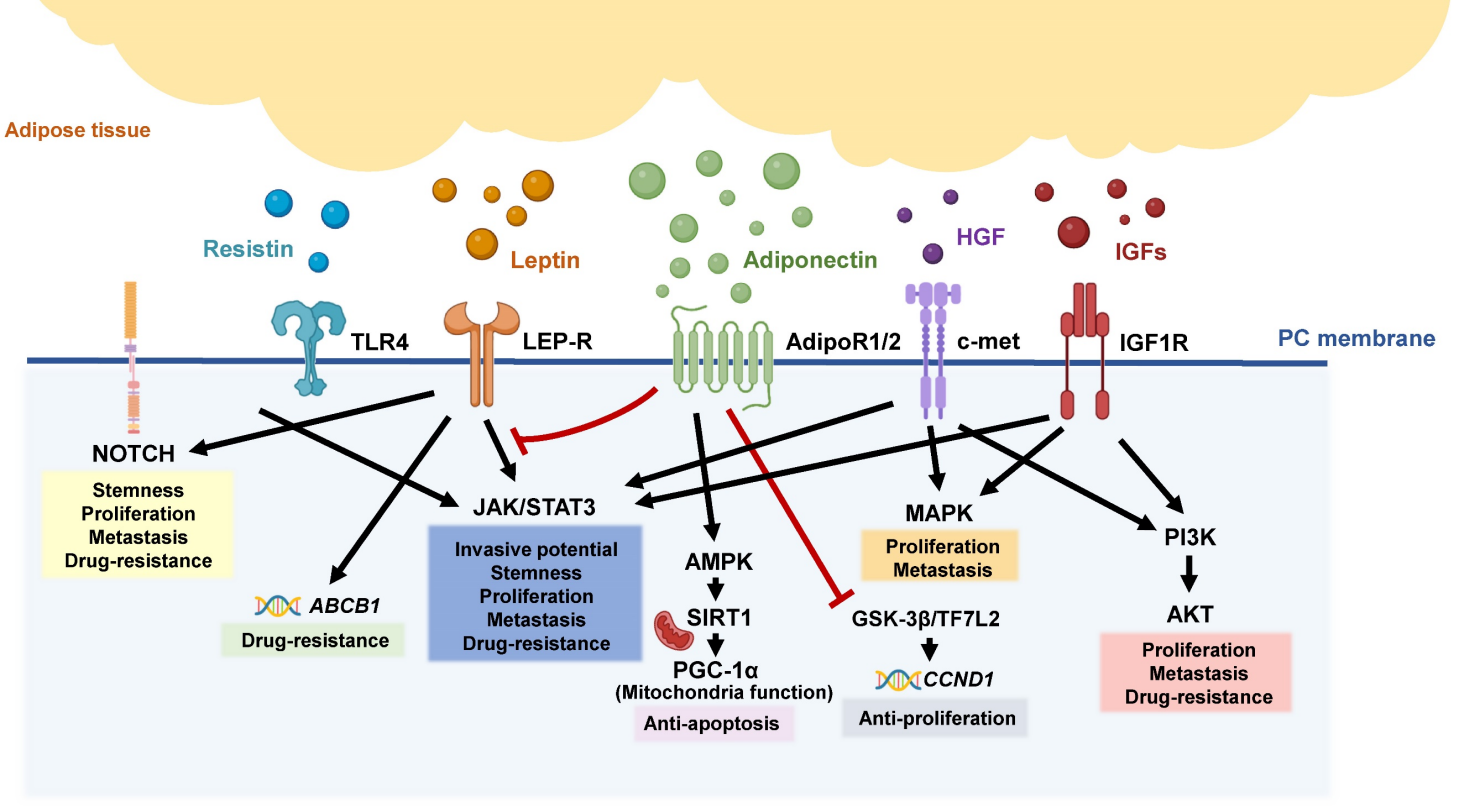
** Adipokine-related pathways in pancreatic tumorigeneses.** Various adipokines are released from inflamed and dysfunctional adipose tissue, triggering different signal pathways involved in the aggressive behaviors of pancreatic tumors, including the NOTCH, JAK/STAT, MAPK, and PI3K/AKT pathways.

During PC tumorigenesis, the high levels of pro-inflammatory cytokines in the TME bring adipose tissue to an inflammatory state, causing tissue fibrosis and even adipocyte death 15, 84-86. Moreover, adipocytes stimulated by PC can secrete lipocalin 2 (Lcn2) to facilitate muscle atrophy and fibrosis. The secretion of Lcn2 is involved in the development of cachexia, a condition characterized by severe tissue wasting, which is implicated in the prognosis of PC patients 87, 88. Above evidence points out the aberrant endocrine function of adipose tissue, which contributes to immune suppression and tumor expansion, thereby potentially impacting patient outcomes. In the following sections, we will discuss the dysregulation of adipokines and their association with PC progression (**Figure 2**).

#### 3.2.1. Leptin

Leptin is a key hormone that regulates appetite and food intake, with additional functions involved in immune response and angiogenesis 89, 90. Even though leptin shows opposite expression patterns in different cancer types, its gene expression is upregulated in response to TNF-α and IL-1 stimulation 91-94, which are elevated in the serum of PC patients and correlated with poor prognosis 95-97. Leptin facilitates the proliferation of human PC AsPC-1, MIA PaCa-2, and BxPC-3 cells through activation of the JAK2/STAT3 pathway. This pathway also contributes to cancer invasion by producing matrix metalloproteinase (MMP)-13 for tissue remodeling 98, 99. In addition, leptin promotes the expression of cancer stem cell markers CD24, CD44, and ESA, with increasing tumor-sphere sizes and numbers by a NOTCH-dependent signal 100. Both STAT3 and NOTCH pathways are vital to gemcitabine-resistance during PC development 101, 102. Indeed, leptin protects PC cells from the cytotoxicity of 5-FU by inducing drug efflux proteins, including ATP binding cassette subfamily B member 1 (ABCB1). This results in an upregulation of anti-apoptotic proteins, including Bcl-xL, and receptor interacting protein (RIP), while inhibiting the degradation of Poly ADP-ribose Polymerase (PARP) and activation of caspase-3 100, 103-105. These findings highlight the important role of leptin in PC progression.

Although the effect of leptin on noncancerous cells within the pancreatic TME is poorly understood, previous research has revealed the roles of leptin in regulating both innate immunity and adaptive immunity. An *in vitro* study has demonstrated that short-term leptin treatment increases IFN-γ secretion and cytotoxicity of NK cells. However, prolonged leptin stimulation can lead to decreased proliferation and immune function of NK cells 106, indicating the opposite effect of leptin on NK development. Both mature and immature DCs express leptin receptors (LEPR), and leptin signal reception promotes their activation and subsequent release of pro-inflammatory cytokines 107. Additionally, leptin stimulates macrophage proliferation in a dose-dependent manner 108. Intriguingly, leptin treatment induces M2-specific surface markers like ARG-1 and Fizz-1 but enhances the production of pro-inflammatory cytokines, including TNF-α, IL-6, IL-1β and CCLs via JAK2/STAT3 mediated pathways 108. Leptin treatment also induces metabolism toward M1-polarization, with increased lipid accumulation and glycolysis observed in macrophages, underscoring the importance of leptin in immune-metabolic regulation 108, 109. Regarding adaptive immunity, CD8^+^ T cells represent the main force against tumor cells. Leptin can also influence the frequency of mitochondrial respiration in CD8^+^ T cells derived from peripheral lymph nodes near the tumor site, thus enhancing CD8^+^ T cells' tumor-killing function in a melanoma model 110. Activation of T cells requires DC-exposed antigens, which can be promoted by leptin as mentioned above, showing that leptin may be a potential immunotherapeutic target during disease progression.

#### 3.2.2. Adiponectin

Adiponectin is the most abundant adipokine secreted by mature adipocytes and possesses insulin-sensitizing and anti-inflammatory abilities 111. Adiponectin seems to play a dual role in PC progression. Adiponectin treatment triggers glycogen synthase kinase-3 beta (GSK-3β)-dependent β-catenin degradation through activation of its receptor AdipoR1/AdipoR2. Loss of β-catenin restricts the function of its coregulator transcription Factor 7 Like 2, which reduces their downstream target cyclin D1 expression and proliferative attenuation in human PC BxPC-3 and CFPAC-1 cells 112. This study supports the anti-tumor role of adiponectin. However, another report showed that adiponectin promotes PC progression by inhibiting apoptosis via the activation of AMPK/Sirt1/PGC-1α signaling 113. Blockade of adiponectin signals through adiporeceptor agonists, which act on both AdipoR1 and AdipoR2, inhibits leptin-mediated STAT3 activation and AMPK activity, ultimately reducing colony formation ability and promoting apoptosis in PC cells 114.

Previous clinical studies have also found that patients with PC have lower plasma adiponectin levels 115, which matches the report showing that adipose tissue expressed less adiponectin under long-term exposure to pro-inflammatory cytokines 116. However, another clinical study detected a higher level of adiponectin in PC patients 117 while a case-control study identified higher expression levels of adiponectin receptors AdipoR1 and AdipoR2 on pancreatic tumors 118. These contradictory results may be attributed to the fact that adiponectin levels were measured at only one time point in these clinical studies. The levels and function of adiponectin may be dynamic during PC progression, and a time-lapse study is needed to understand the exact role of adiponectin in PC.

#### 3.2.3. Resistin

Resistin is originally secreted from adipose tissue and takes part in obesity, inflammation, and insulin resistance. A clinical study indicated that patients with resistin-positive pancreatic tumors displayed shorter relapse-free survival times 119, 120. After binding to adenylyl cyclase‐associated protein 1 and toll‐like receptor 4 (TLR4), resistin activates STAT3 to promote gemcitabine-resistance via modulating cell cycle from arresting S phase enter into G2-M phase in MIA PaCa‐2 and SW1990 cells 120. These results suggest that resistin released from adipose tissue may support the survival of cancer cells.

#### 3.2.4. Hepatocyte growth factor (HGF)

HGF has been well known to promote epithelial cell proliferation and cell motility 121. HGF produced by adipocytes can interacts with its receptors c-mesenchymal-epithelial transition factor (c-Met) on PC cells to promote the proliferation and migration of Panc02 and TGP-47 murine cells through activation of downstream MAPK, PI3K/AKT, and STAT3 signaling 122-124. c-Met upregulation and elevated circulating HGF have been found in locally advanced PC and are associated with poor clinical outcomes 125. HGF/c-Met activation also provides chemoprotection against gemcitabine during *Kras^G12D/+^*-driven pancreatic intraepithelial neoplasia development *in vivo* 126. HGF secreted from PSCs has been demonstrated to mediate tissue remodeling, tumor invasion, and metastasis in PC. This effect can be further exacerbated by the excessive release of HGF from dysfunctional adipocytes.

#### 3.2.5. Insulin-like growth factors (IGFs)

IGFs have two isoforms, IGF1 and IGF2, and are produced by the liver and adipose tissue. IGFs participate in adipocyte differentiation and blood glucose regulation, and they have been reported as cancer risk factors in various tumor types 127. During PC progression, inflammation, and insulin resistance promote IGF secretion from adipose tissue 128, 129. IGF-1 protects PC cells from gemcitabine and Nab-Paclitaxel through activating AKT, which can be diminished by specific antibody blockade with their receptor IGF-1R 130. IGF-1R signaling can also promote the proliferation and metastasis of HPAC and PANC-1 cells through the PI3K/PTEN/AKT, MAPK, and JAK/STAT pathways 131-133. Inhibition of AKT is able to decrease IGF-1R expression and reduce PC cell growth 134.

#### 3.2.6. Proinflammatory cytokines

Normally, resident macrophages in adipose tissue produce anti-inflammatory factors to maintain an appropriate environment for regular adipose tissue function. However, during cancer progression, systemic inflammation activates macrophages in adipose tissue, leading to the secretion of inflammatory cytokines from adipocytes, which in turn results in immune cell infiltration, intensifying the immune response in adipose tissue 135, 136. Among these cytokines, IL-1β, IL-6, and TNF-α are well-known to mediate PC progression by promoting desmoplasia, proliferation, metastasis, immunosuppression, and chemoresistance 137-140. A previous study on secretome has shown that omental adipocytes secrete IL-1β, IL-6, and TGF-β1 into pancreatic the TME, promoting the growth, metastasis, and chemoresistance of PC cells 141. Another study has demonstrated that CXCL14 secreted from thermogenetically activated beige/brown adipocytes promotes the recruitment of macrophages and M2 polarization in high-fat diet-induced obese animal models, indicating that the browning of adipocytes may also contribute to immunosuppression in PC 142. These studies offer clues that adipose tissue enhances systemic inflammation response by secreting a variety of pro-inflammatory cytokines, which promote cancer growth and survival and can even intensify fat wasting via autocrine effects. This creates a vicious circle that impairs adipose tissue function and exacerbates the pathological processes.

### 3.3. Adipocyte plasticity in PC development

**Figure 3:**
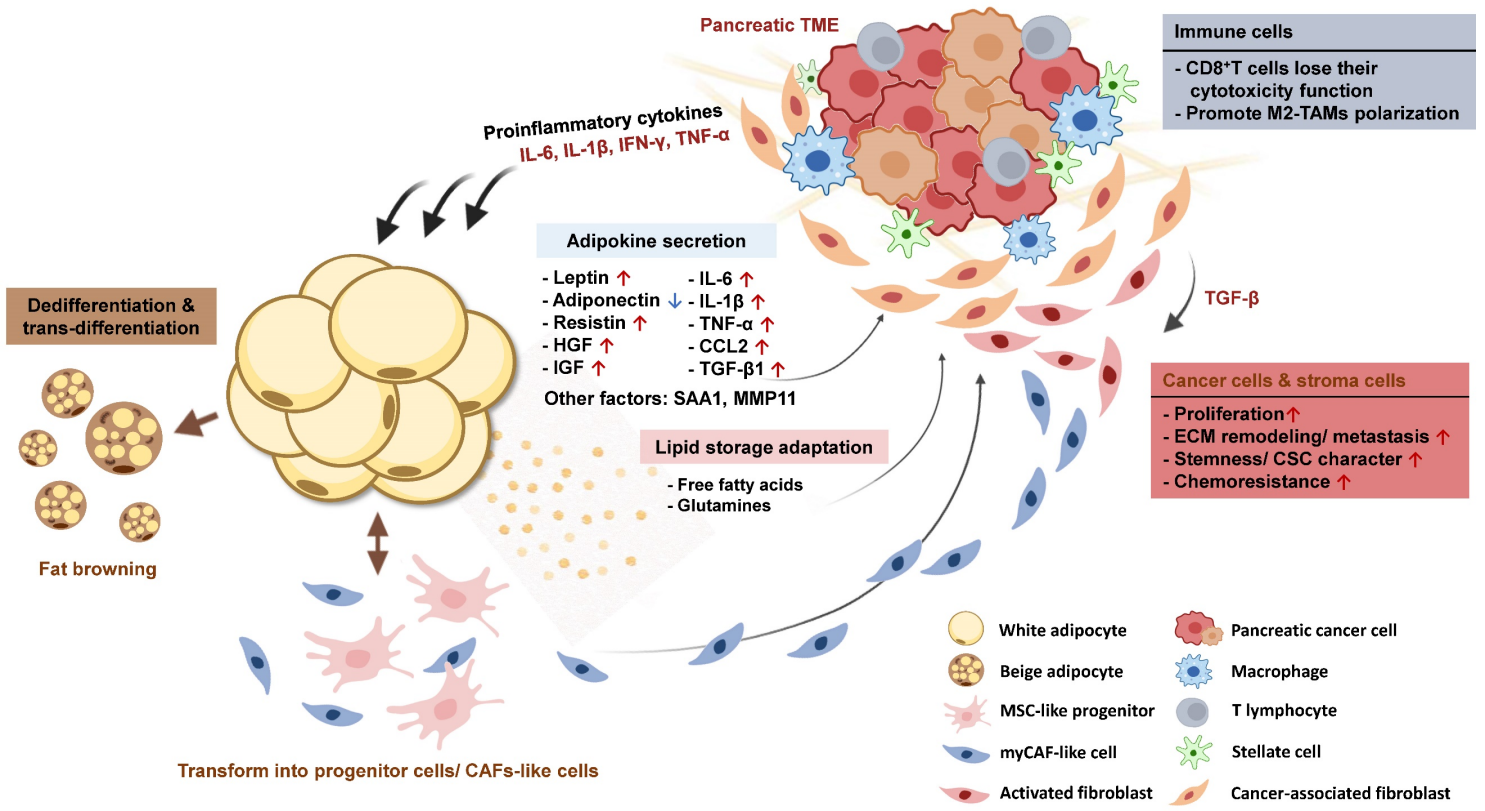
** Interaction between pancreatic tumors and adjacent adipose tissue promotes adipose tissue dysfunction and cancer progression.** Dysfunctional adipocytes support PC growth through various secreting factors. Long-term stimulation of pro-inflammatory cytokines also transforms white adipocytes into beige adipocytes, MSC-like progenitor cells, or CAF-like cell types that participate in ECM remodeling in PC.

Numerous signal pathways that sustain the normal function of adipocytes are interfered with by stimulation of cytokines derived from the pancreatic TME or adipose tissue itself, leading to altered expression of adipogenic and metabolic genes. Over time, adipocytes gradually lose their specific characteristics acquired during their development and undergo phenotypic changes, transitioning into progenitor-like cell types or directly trans-differentiating into pro-tumor fibroblasts. In this section, we will discuss the phenotypic changes observed in adipocytes following their interaction with PC cells and elucidate how these dedifferentiated adipocytes contribute to the progression of PC (**Figure 3**).

#### 3.3.1. Morphological change and impaired function of adipocytes during PC progression

During the progression of PC, adipocytes undergo distinct morphological changes. When interacting with PC cells, adipocytes lose their lipid stores and transit into fibroblast-like cells 143, 144. Gene expression profiling studies have revealed that co-culturing adipocytes with PANC-1 cells resulted in a significant downregulation of genes associated with adipogenesis, lipogenesis, and TG storage in mature adipocytes. Additionally, in the presence of PC cells, mature adipocytes lose their specific markers including adiponectin, leptin, resistin, and fatty acid-binding protein 4 (FABP4), while acquiring fibroblast markers like α-smooth muscle actin (α-SMA), S100 Calcium Binding Protein A4 (S100A4), FSP-1, plasminogen activator inhibitor-1, MMP-9 and MMP-11 143, 144. These findings suggest that PC cells can induce fibrogenic changes in adipocytes.

In addition to adipocytes, skeletal and smooth muscle in patients of PDAC exhibit increased collagen content, which has been associated with lymph node metastasis 88, 145, 146. This muscle fibrosis appears to be influenced by the function of TGF-β, which is known to be released by TME and dysfunctional adipose tissue. The collaborative effect of fibrosis in both adipose tissue and muscle, along with the enhanced ECM deposition, creates a favorable environment for the expansion of PC.

#### 3.3.2. Mechanism involved in adipocyte dedifferentiation

In the absence of adipogenic gene expression, particularly PPAR-γ, adipocytes undergo a fibrotic pathway leading to cellular reprogramming, which transforms adipocytes into preadipocytes, mesenchymal progenitor cells, or myofibroblasts 147. The Wnt pathway is one of the important mechanisms in mediating adipocyte dedifferentiation. IL-6 and TGF-β derived from the PC TME induce Wnt5a expression in mature adipocytes, triggering the downstream signaling involving c-Jun/activator protein-1 (AP-1) complex for the transcription of genes associated with fibroblast transformation and MMP proteins 148, 149. In addition to adopting fibroblast phenotype, adipocytes can transit to acquire stem cell characteristics. After being co-cultured with MIA PaCa-2 cells, mature adipocytes exhibited significant induction of genes associated with pluripotent stem cells, such as *Oct3/4*, *Klf4*, *c-Myc,* and *Sox2* 149. Another study has also suggested that dedifferentiated fat cells possess a multilineage potential to differentiate into various cell types within the mesenchymal lineage, including adipocytes, chondrocytes, and osteoblasts, under appropriate stimulation 150.

Collectively, the stimulation of pro-inflammatory cytokines or other factors secreted from the pancreatic TME can trigger the trans-differentiation of adipocytes into mesenchymal lineage cells or fibroblast-like cells. Adipocytes in close contact with cancer cells may progressively transit into cancer-associated fibroblasts (CAFs), which contribute to fibrosis and tissue remodeling within the TME and further intensify the chemoresistance and metastatic potential of PC.

### 3.4. Interaction between adipose tissue and pancreatic TME

Obesity and other metabolic syndromes have been linked to the progression of PC. In addition to the influence of remote subcutaneous fat, fat tissue adjacent to the tumor also plays a vital role in cancer development. In the KPC transgenic mouse model, a high-fat diet significantly increases the percentages of intra-tumoral adipocytes and the incidence of distant metastasis compared with a standard diet 65. Clinical research has depicted that PC tumors with peripancreatic fat infiltration are typically associated with advanced cancer stage, locoregional recurrence, and reduced overall survival 24. Increased fat accumulation in acinar cells also leads to the extra release of pro-inflammatory cytokines, which recruit immune cells and modulate immune responses in the pancreatic TME 26, 27. In this section, we will focus on how adjacent adipose tissue interacts with the TME to exacerbate PC aggressiveness.

#### 3.4.1. The effect of mature adipocytes on PC progression

Conditioned medium (CM) collected from omental fat promotes the proliferation, invasion, migration, and gemcitabine resistance of PC cells 65. The results from xenograft nude mouse models of PC have shown that the CM of adipocytes had a higher angiogenic potential 141. In addition, the secretome analysis of omental CM identified 157 cancer progression-associated proteins, with IL-6, IL-1β, and TGF-β as the key upstream mediators. *ANGPTL4* and *SPP1*, encoding angiopoietin-like 4 and osteopontin respectively, were found to be upregulated in CM-treated PC cells. Both are ECM-associated glycoproteins involved in stroma structure remodeling for tumor metastasis 141, 151. These findings support that an adipose-rich environment may boost PC development.

Besides the uni-directional signal from adipocytes, the crosstalk between adipocytes and PC cells also brings benefits for tumor growth. Previous studies have shown that adipocytes increase serum amyloid A1 (SAA1) expression in PC, which mediates PC cell migration by upregulating metastasis-related proteins N-cadherin, Snail, and Slug 144. SAA1 expression is clinically associated with a poor prognosis of PC and plays an important role in 5-Fluorouracil (5-FU) resistance and stroma formation 144, 152. Because SAA1 has been demonstrated to activate NF-κB and proinflammatory genes 153, 154, it may further facilitate the inflammation response in the TME. These findings suggest the noticeable role of adipocyte-induced SAA1 in PC tumor progression.

The interaction between adipocytes and PC cells also enhances the expression of MMP-11 in PC cells, which has been reported to inhibit apoptosis and is capable of degrading ECM components for tumor invasion or migration 155. MMP-11 accelerates the cell cycle of PC cells by upregulating cyclin-dependent kinase 4 and cyclin D1 156. Of note, adipocytes can also secrete MMP-11 to affect tumor growth when they are nearby due to peripancreatic fat infiltration or PC metastasis into adipose tissue 157. Not only mature adipocytes but also the resident progenitors in adipose tissue, contribute to PC development.

#### 3.4.2. The role of adipose tissue-derived stem cells in PC

Adipose tissue-derived stem cells (ADSCs) are mesenchymal stem cells that stay in the stromal vascular fraction of adipose tissue and are capable of self-renewal as well as multipotent differentiation. ADSCs near the tumor site can easily interact with surrounding cancer cells. ADSCs excrete stromal cell-derived factor-1 (SDF-1) into the interstitial space and turn on CXC chemokine receptor 4 (CXCR4) signaling in PC cells, consequently triggering the proliferation, invasion, and migration of PC 158. SDF-1/CXCR4 signaling has also been reported to regulate apoptosis in cancer cells and participates in tumor angiogenesis by recruiting endothelial progenitor cells 159-161. Extracellular vesicles originating from ADSCs have been found to carry the long non-coding RNA (lncRNA) NEAT1, which promotes PC progression by facilitating proliferation, upregulating stemness-related genes, and enhancing resistance to gemcitabine. These effects are achieved through the suppression of miR-491-5p expression. Moreover, the elevated expression of the EMT-related protein Snail, along with the downregulation of suppressor of cytokine signaling (SOCS) proteins, which act as negative regulators of the JAK/STAT3 signaling pathway, can further contribute to the progression of PC 162. However, there is also conflicting evidence about the role of ADSCs in cancer progression. A previous study reported that conditioned medium collected from primary ADSCs induced cell cycle delay by inhibiting CDK4 and cyclinD1, simultaneously promoting necrosis of PC cells. These effects ultimately lead to a reduction in tumor volume in a Capan-1 subcutaneous injection model 163.

In addition to the impact of secreted factors from ADSCs on PC growth, the inherent pluripotency of ADSCs also enables them to undergo phenotypic changes that promote PC development. When exposed to external stimuli, ADSCs can differentiate into various cell types. Specifically, TGF-β released by pancreatic tumors activates ADSCs, leading to the downregulation of S100A4 expression and their subsequent transformation into CAF-like cells. These transformed cells acquire an enhanced ability to produce ECM components that support PC cell migration 164. Distinct co‐culture conditions also determine the fate of ADSCs. When ADSCs come directly in contact with Capan‐1 cells, they differentiate into myoblastic CAFs (myCAFs) and inflammatory CAFs (iCAFs). When ADSCs were co-cultured with PC indirectly through the trans-well system, the iCAF phenotype was induced 165. iCAFs with high expression of *IL6*, *CXCL1*, and* LIF* genes are characterized by a secretory phenotype and are able to stimulate STAT3 activation in PC cells. myCAFs with high *ACTA2* (α-SMA) and *TPM1* expression are usually adjacent to cancer cells and contribute to ECM production 165-167.

Given the high potential of ADSCs transiting into pro-tumor CAFs, targeting the factors and mechanisms involved in this process can potentially suppress the development and progression of PC. A single-cell analysis from patients' samples reveals the gene expression pattern during ADSCs-CAFs conversion. *APOD* is the representative marker of the ADSCs population, together with other genes like *CFD*, *CXCL12*, *MGP,* and *PTGDS* expressed predominantly. During transformation, *SFRP4*, a Wnt pathway regulator, and *RARRES1*, which is known for regulating the proliferation and differentiation of ADSCs were both highly expressed. At the same time, the levels of PLA2G2A proteins were elevated during this transition process. PLA2G2A participates in the hydrolysis of phospholipids, leading to the production of FFAs that provide additional energy resources for cancer metastasis. At the end of the transition, ADSCs lose the expression of *SFRP4* and *RARRES1* and turn into *COL11A1*-expressing CAFs 168. This time-scale experiment identifies the essential regulators during ADSC-CAF transition, inhibition of this pathway may be able to reduce CAF populations and their related desmoplasia.

## 4. Targeting adipose tissue for pancreatic cancer treatment

Emerging evidence underscores the vital role of adipose tissue in the development of PC. There is increasing interest in exploring innovative approaches to target dysfunctional adipose tissue, intending to reduce the aggressiveness of tumors and overcome treatment resistance in PC. Strategies such as blocking catabolic pathways, regulating the production of specific molecules known as adipokines and their downstream effects, or preventing the transformation of adipocytes show great promise in this regard. In this section, we provide a summary of the current applications of anti-pancreatic tumor reagents that target different functions of adipose tissue and their impact on the development of PC (**Table 1**). By understanding the role of adipose tissue in PC progression, novel therapeutic approaches can be explored to effectively counteract this disease.

### 4.1. Receptor/downstream signaling blockade

Adipocytes upregulate pro-survival and pro-metastasis signaling in PC cells, promoting their growth through direct contact or secretion of adipokines. Numerous *in vitro* and *in vivo* studies have revealed that targeting these pathways could successfully reduce PC growth.

Clinical studies have demonstrated the correlation between high serum leptin and the risk of PC 169, 170. The protumor functions of leptins include promoting PC proliferation, tumor sphere formation, cell cycle progression, and chemoresistance through JAK/STAT or NOTCH signaling pathways 100, 103-105. The activation of Notch signaling by γ-secretase, which cleaves the Notch 1 intracellular domain, has been associated with increased proliferation, migration, invasion, and drug resistance in PC cells 171, 172. Blockade of the Notch1 pathway with a leptin receptor antagonist or γ-secretase inhibitor enhances tumor sensitivity to gemcitabine and 5-FU and reduces pancreatic tumor size 100, 102, 104, 173. The JAK/STAT pathway participates in tumorigenesis and regulates the expression of programmed death ligand-1 (PD-L1) that suppress the immune response in PC cells 174-176. STAT3 inhibition by a competitive small-molecule inhibitor of JAK1/2 kinase, such as AZD1480, sensitizes PC cells to gemcitabine 101. Therefore, targeting leptin and its downstream signaling pathways represent a conceivable strategy for suppressing pancreatic tumorigenesis.

SAA1 secreted from dedifferentiated adipocytes has been implicated in promoting migration and chemoresistance to 5-FU in PC cells 144. Studies conducted in a glioblastoma model have demonstrated that SAA1 provides chemo-protection by activating AKT and downstream anti-apoptotic proteins such as BCL-2 and BAX 177. The expression of SAA1 is regulated by H3K27 acetylation on its enhancer region, and transcription factors NF-κB and STAT3 also bind to the SAA1 promoter region. Treatment with JQ1 and mivebresib, inhibitors of the BET bromodomain involved in lysine acetylation, has been shown to effectively reduce the expression of SAA1 178. These findings suggest that targeting SAA1 through epigenetic modification in dedifferentiated adipocytes represents a potential therapeutic strategy for cancer.

Adipocyte-derived HGF enhances PC cell growth and gemcitabine-resistance through its interaction with the c-Met receptor. The HGF/c-Met pathway is upregulated in PC and is involved in the interaction with stromal PSCs 126. Blockade of the HGF/c-Met signaling axis by either the ligand inhibitor rilotumumab or the receptor agonist Compound A, in combination with gemcitabine, has been found to significantly reduce pancreatic tumor size and metastasis 123. A similar inhibitory effect has also been observed during the early development of PC in orthotopic mouse models 179, suggesting that targeting HGF/c-Met signaling to abolish the communication between PC cells and PSCs provides a promising approach for PC treatment.

Proinflammatory cytokines originating from both the pancreatic TME and inflamed adipose tissue facilitate tumor progression. Targeting the signal pathways of these cytokines is crucial for PC treatment. TGF-β exhibits distinct roles in both pro-tumorigenic and tumor-suppressive processes during PC progression, depending on the specific tumor stage and microenvironment. It exerts suppressive effects on tumor-induced inflammation and early carcinogenesis 180. However, its downstream signaling pathway also inhibits the activation of CD8^+^ T cells 181. Various strategies have been employed to disrupt TGF-β signaling, including RNA interference (RNAi), neutralizing antibodies, and small molecule inhibitors, which have shown promising results in animal models and clinical trials with improved survival rates 181-183. In addition to TGF-β, proinflammatory cytokines such as TNF-α, IL-6, and IL-1β released from both the PC TME and inflamed adipose tissue have been found to cause adipose wasting. Blocking of these cytokines and their associated signaling pathway has demonstrated efficacy in slowing PC development and preserving adipose function. For instance, inhibition of the IL-6/STAT3 signal pathway using anti-IL-6 receptor antibodies has been successfully in reducing tumor growth, local recurrence, and distant metastasis 184. Similarly, the blockade of TNF-α signaling has shown anti-cancer effects, mainly by reducing tumor volume and inhibiting liver metastasis 96. Furthermore, suppression of the IL-1β pathway has been shown to preserve gemcitabine-mediated cancer cell apoptosis in AsPC-1 orthotopic xenograft models 185.

### 4.2. Modulation of metabolic functions in TME components

Overactivated lipolysis induced by proinflammatory cytokines during PC progression leads to the release of FFAs into the circulation or surrounding tumor area. These FFAs can be taken up and accumulated in the pancreatic TME, which not only serves as a source of nutrients for tumor growth but also induces CD8^+^ T cell dysfunction and M2 macrophage polarization that impair anti-tumor immunity. To combat these effects, it is crucial to suppress PC-induced fat wasting and reduce lipid uptake by the TME. Inhibiting the uptake of lipids by targeting scavenger receptor CD36 through genetic depletion, gene silencing, or irreversible inhibitor sulfosuccinimidyl oleate (SSO) treatment has been demonstrated to maintain immune system functions, prevent gemcitabine resistance, and inhibit PC metastasis 72, 77, 186-188.

In addition to blocking lipid influx, disturbing the metabolism of PC also has a significant impact on tumor growth. Pancreatic tumors with high ATGL levels show increased desmoplasia 189, while PC implantation in adipose tissue switches its metabolism from glycolysis to FAO 190, showing the significant role of fatty acid metabolism in PC progression. Fatty acid oxidation inhibition with trimetazidine reduced ATP production in human PC cell lines remarkably 191. Targeting FAO by inhibiting the transport of fatty acids into mitochondria with etomoxir has been shown to reduce cell proliferation and promote apoptosis in PC cells under treatment with an anti-angiogenesis reagent.

On the other hand, targeting the upstream regulator of lipolysis is an effective way to prevent fat wasting during PC progression. Administration of anit-IL-6 receptor antibodies or genetic knockout of IL-6 prohibits lipid storage loss from adipose tissue 52, 192. Treatment with inhibitors against JAK, a downstream kinase of IL-6, prevented adipose tissue loss and prolonged the lifespan of tumor-bearing mice 193. This evidence highlights the importance of blocking lipid breakdown or cellular uptake as a critical strategy for suppressing tumor growth. However, it remains uncertain whether the observed antitumor effects in these studies are solely attributed to the inhibition of fat-wasting, as the pathways involved in lipolysis are not exclusive to adipose tissue.

### 4.3. Inhibition of adipocyte dedifferentiation

Mature adipocytes can transit into CAF-like cells under chronic cytokine stimulation to exacerbate desmoplasia, metastasis, and chemo-resistance of PC. It is known that adipocyte dedifferentiation initiates activation of Notch signaling to cut down fatty acid synthesis and PPAR-γ activity that sustains adipogenesis. Wnt5a signaling induced by TGF-β and IL-6 promotes MMPs production and the expression of fibrotic genes in the adipocytes. Treatment with rosiglitazone, a synthetic ligand for PPAR-γ, has successfully reversed the dedifferentiation of adipocytes 148. Similarly, treatment with IWP2, an inhibitor of Wnt ligand secretion, has been shown to preserve the normal morphology of mature adipocytes. Additionally, the use of Wnt-5a neutralizing antibodies as well as a Wnt antagonist, such as secreted frizzled related-protein 5, has also been found to decelerate the transition of adipocytes 149. A study conducted on mouse models of breast cancer revealed a significant reduction in tumor growth when dedifferentiated adipocytes were absent 194. These findings provide compelling evidence that targeting the plasticity of adipocyte differentiation holds promise for suppressing the progression of PC.

## 5. Conclusions and Perspectives

As the significance of lipid metabolism in tumor progression becomes more prominent, an increasing number of studies are now focusing on the role of adipose tissue in the development of PC, especially in obese subjects. Growing evidence suggests a strong connection between adipose tissue and aggressive PC, characterized by enhanced metastasis and chemoresistance. Long-term exposure to proinflammatory cytokines secreted from the pancreatic TME triggers the release of excessive lipid resources and abnormal adipokines from dysfunctional adipose tissue, providing a supportive environment for cancer cell proliferation and the establishment of a tumor-promoting TME. The aberrant transition of mature adipocytes into a cell type exhibiting myCAF characteristics gives rise to severe desmoplasia in the pancreatic TME. Cytokines and growth factors produced by these cells further stimulate the proliferation, invasion, migration, and chemoprotection of PC. Thus, disrupting the communication between adipose tissue and PC cells holds great promise for improving the efficacy of antineoplastic treatments. Indeed, *in vivo* studies have demonstrated significant advancements in anti-pancreatic tumor progression and improving survival by employing this approach. Combining targeted anti-adipokine reagents or neutralizing antibodies with chemotherapy drugs has proven successful in overcoming chemoresistance mediated by dysfunctional adipocytes. These findings provide valuable insights and a hopeful outlook for overcoming cancer drug resistance in clinical treatment. They offer valuable guidance for future therapeutic strategies, paving the way for innovative directions in the field of cancer treatment. However, there remains a need to better understand the impact of adipose tissue on pancreatic tumor expansion and the mechanisms underlying the transition of adipocytes.

Most studies investigating the involvement of adipose tissue in PC progression have utilized obesity as their disease model. However, emerging evidence suggests that adipose tissue adjacent to the pancreas can promote locoregional recurrence and result in unfavorable clinical outcomes, even in the absence of metabolic disorders. In animal models of cachexia, the loss and remodeling of adipose tissue have been observed during PC development, showing that while obesity can be a contributing factor, it is not a necessary condition for disease progression. Chronic inflammation induced by adipose tissue itself during obesity creates a metabolic imbalance and unhealthy immune response that may not accurately reflect the conditions in patients without metabolic diseases. Therefore, further investigation is needed to understand the complicated communication between adipose tissue and PC. Additionally, different adipose tissue depots exhibit heterogeneous responses to physiological stimulation. For instance, epididymal and mesenteric adipose tissue display a higher inflammation status compared to subcutaneous depots during tumor progression. This indicates that the distance between adipose tissue and the tumor may result in varying degrees of impact. While most studies have focused on peritoneal fat in PC analyses, it is important to consider that proinflammatory cytokines released from TME may influence all fat depots through the systemic circulation. Exploring whether other adipose tissue depots exhibit similar pro-tumor effects as peritoneal fat is an interesting area for future investigations. Moreover, limited attention has been given to the direct effects of adipokines on immune cells rather than cancer cells in PC models. The immune escape continues to pose a significant challenge in the treatment of PC, and exploring the potential role of adipokines in the development of treatment resistance in PC cells could yield valuable insights for overcoming this difficulty. Future research focusing on the direct impact of adipokines on immune cells may uncover novel strategies to enhance immune responses and improve treatment outcomes in PC.

Nowadays, increasing evidence reveal the connection between adipose tissue and the clinical outcomes of PC, highlighting adipose tissue as a promising target for therapeutic interventions. However, it is important to note that most intervention methods targeting adipose-tumor interactions have primarily been tested *in vitro*. The lack of *in vivo* evaluation hinders better assessment of their efficacy and safety, which is essential before clinical translation. Although the journey towards the clinical use and approval of these therapeutics may be long, acquiring a more comprehensive understanding of the molecular mechanisms through which adipose tissue promotes pancreatic tumors and associated wasting symptoms will undoubtedly contribute to the development of more effective anti-cancer strategies.

## Figures and Tables

**Table 1:** Inhibition of the interaction between PC and adipose tissue by different mechanisms.

Inhibiting adipokines downstream signal pathways					
Inhibitor name	Major molecular target	Experiment model	Results	Notes	References
LPrA2 (Leptin receptor antagonist)	Leptin-Notch signal	MIA PaCa-2 xenografts in Nude CD1 nu/nu mice	Delayed tumor onset Reduced tumor volume Decreased cancer stem cell marker expression	LPrA2 was delivered by iron oxide nanoparticles	100
DAPT (γ-Secretase Inhibitor)	Notch signal	Human pancreatic cancer cell lines, BxPC-3, PANC-1, and MIA PaCa-2	Deceased number and size of tumor spheres Enhanced 5-FU sensitivity through increasing caspase-3 activity (apoptosis induction)	DAPT+5-FU	100, 104
RO4929097 (γ-Secretase Inhibitor)	Notch signal	Human tumor-sphere implant in NOD/SCID mice	Reduced cancer proliferation and tumor size Declined cancer stem cell population (ESA^+^/ CD44^+^/ CD24^+^)	RO4929097+Gemcitabine	173
PF-03084014 (γ-Secretase Inhibitor)	Notch signal	Patient-derived PC xenografts in athymic nude mice	Enhanced anti-tumor effect of gemcitabine Preventing lung and liver metastasis Suppressed tumor proliferation Reduced angiogenesis in pancreatic tumor	PF-03084014+Gemcitabine	102
AZD1480	JAK/STAT3 signal	PANC-1 xenografts in Fox1-nu/nu mice	Reduced tumor size Improved mice survival Depleted desmoplastic stroma Enhanced gemcitabine delivery in tumor	AZD1480+Gemcitabine	101
Rilotumumab (human anti-HGF mAb^a^)/ Compound A (Blocking c-met activation)	HGF-c-Met signal	AsPC-1 orthotopically inoculates in BALBc nu/nu mice	Increased gemcitabine-induced cancer cell apoptosis Reduced tumor size and metastasis events Decreased the expression of stemness markers	A triple combination treatment of Rilotumumab, compound A, and Gemcitabine shows the highest anti-cancer effect.	123
Galunisertib	TGF-β/Smad2 signal	PANC-1 human PC cells	Reduction in the population of CD25^+^FOXP3^+^ T_reg_ cells Reduced stroma by suppressing the proliferation of epithelial cells	Transgenic mice with MT-*TGFBR2^DN^* or globally *Tgfbr1*^+/-^ deficiency^b^ exhibit improved stromal fibrosis and restored CD8^+^ T cell cytotoxicity during PC development.	181
2G8 (TGFβR2 inhibitor)	TGF-β/Smad2/4 signal	Transgenic model *KIC*^c^ mice and *KPC*^d^ mice	Decreased IL-6 secretion from stroma Promoting M1-like macrophages, NK cells, and cytotoxic CD8^+^ T cell activation	2G8 treatment shows decreased tumor proliferation and improved survival only in TGFβR2-deficient pancreatic tumors.	182
Tocilizumab (anti-IL-6 receptor antibody)	IL-6/STAT3 signal	Colo357 and PancTuI cells orthotopic xenografts in SCID/bg mice	Reduced tumor weight and proliferation Decreased local recurrence and distant metastasis	The combination treatment of Tocilizumab and Gemcitabine did not show a significant additive effect.	184
Etanercept/ Infliximab	TNF-α signal	Colo357, BxPC-3, and PancTuI cells orthotopic xenografts in SCID/bg mice	Reduced tumor volume and liver metastasis	Etanercept and Infliximab have been plunged into clinical trials of PC treatment but failed to enhance Gemcitabine efficiency.	96, 195, 196
Anakinra (human IL-1 receptor antagonist)	IL-1/NF-κB signal	AsPC-1 orthotopic xenograft in athymic nude mice	Reduced tumor size through suppressing proliferation Enhanced Gemcitabine sensitivity	The combination of Gemcitabine and Anakinra reduced the migration and induced apoptosis in PC cells.	185
**Suppressing PC progression through metabolic function**					
Etomoxir	Inhibition of carnitine palmitoyl transferase 1A (CPT1)/ Fatty acid oxidation	Panc02 syngeneic model with subcutaneously implantation into C57BL/6 mouse	Combination treatment of Etomoxir and anti-VEGF significantly reduced proliferation and enhanced apoptosis in PC	The inhibitory effect on PC proliferation only displays on combination treatment of Etomoxir and anti-VEGF treatment.	190
Trimetazidine	Fatty acid oxidation	MIA PaCa-2 and SNU-324 human PC cells	Trimetazidine treatment reduced ATP production and PC proliferation	PC produces ATP through FAO more than glycolysis.	191
**Targeting adipocyte dedifferentiation**					
Anti-Wnt5a antibody/SFRP-5	Wnt/β-catenin Wnt5a/c-Jun/AP-1	*In vitro* study of MIA PaCa-2 PC cells with 3T3-L1 adipocytes	Inhibition of the Wnt5a signal partially sustains adipocyte morphology and lipid storage	MIA PaCa-2 cells induced the transition of adipocytes into fibroblast-like phenotype.	149

^a^mAb: monoclonal antibody; ^b^MT-*TGFB2*^DN^: TGF-β suppression through expressing dominant-negative *TGFBR2* in epithelial cells; MT, metallothionein 1 promoter, allows gene modification in tissue that rich in cation, including pancreas, gastrointestinal tract, and liver. ^c^*KIC* mice: mice with genetical engineer in *Kras^LSL-G12D/+^*; *Cdkn2a^flox/flox^*; *Ptf1a^Cre/+^.*^d^ *KPC* mice with genetical engineer in *Kras^LSL-G12D/+^*; *Trp53^LSL-R172H/+^*; *Ptf1a^Cre/+^* mice.

## Abbreviations

5-FU: 5-fluorouracil
ABCB1: ATP binding cassette subfamily B member 1
ADSCs: adipose-derived mesenchymal stem cells
AMPK: AMP activated protein kinase
AP-1: activator protein-1
ATGL: adipose triglyceride lipase
ATP: adenosine triphosphate
BAT: brown adipose tissue
BMI: body mass index
BMP: bone morphogenic protein
C/EBP: CCAAT-enhancer-binding protein
CAFs: cancer-associated fibroblast
CC: cancer cachexia
CCL-2: C-C motif chemokine ligand 2
CM: conditioned medium
c-Met: c-mesenchymal-epithelial transition factor
CXCR4: CXC chemokine receptor 4
DCs: dendritic cells
DGs: diglycerides
ECM: extracellular matrix
FABP4: fatty acid binding protein 4
FAO: fatty acid oxidation
FFAs: free fatty acids
FGFs: fibroblast growth factors
GSK-3β: glycogen synthase kinase-3 beta
HGF: hepatocyte growth factor
HSL: hormone-sensitive lipase
iCAFs: inflammatory CAF
IFN-γ: interferon gamma
IGFs: insulin-like growth factors
IL: interleukin
JAK: janus kinase
JNK: jun N-terminal kinase
KC: *fElas^CreERT^;Kras^G12D/+^*
KPC: *Pdx1-Cre;LSL-Kras^G12D/+^;Trp53^R172H/+^*
Lcn2: lipocalin 2
LEPR: leptin receptors
lncRNA: long non-coding RNA
MAPKs: mitogen-activated protein kinases
MGL: monoglyceride lipase
MMP: matrix metalloproteinase
MSCs: mesenchymal stem cells
myCAFs: myoblastic CAFs
Myf5: myogenic factor-5
NF-κB: nuclear factor kappa B
NK cells: nature killer cells
PARP: poly ADP-ribose polymerase
PC: pancreatic cancer
PGC-1α: PPAR-γ co-activator 1 alpha
PI3K: phosphatidylinositol 3-kinase
PPAR-γ: peroxisome proliferator-activated receptor gamma
PSCs: pancreatic stellate cells
RIP: receptor interacting protein
RNAi: RNA interference
ROS: reactive oxygen species
S100A4: S100 calcium binding protein A4
SAA1: serum amyloid A1
SDF-1: stromal cell-derived factor-1
SOCS: suppressor of cytokine signaling
STAT: signal transducer and activator of transcription
TAMs: tumor-associated macrophages
TG: triglycerides
TGF-β: transforming growth factor beta
TLR4: toll‐like receptor 4
TME: tumor microenvironment
TNF-α: tumor necrosis factor alpha
UCP1: uncoupling protein 1
WAT: white adipose tissue
Wnt: wingless-related integration site
α-SMA: alpha-smooth muscle actin
